# Dual-Mode Integration of a Composite Nanoparticle in PES Membranes: Enhanced Performance and Photocatalytic Potential

**DOI:** 10.3390/nano15141055

**Published:** 2025-07-08

**Authors:** Rund Abu-Zurayk, Nour Alnairat, Haneen Waleed, Aya Khalaf, Duaa Abu-Dalo, Ayat Bozeya, Razan Afaneh

**Affiliations:** 1 Hamdi Mango Center for Scientific Research, The University of Jordan, Amman 11942, Jordan; alnairatnour@gmail.com; 2 Nanotechnology Center, The University of Jordan, Amman 11942, Jordan; 3 Chemical Engineering Department, Jordan University of Science and Technology, Irbid 22110, Jordan; hwmohammad21@eng.just.edu.jo; 4 Allied Sciences Department, Faculty of Arts and Sciences, Al-Ahliyya Amman University, Amman 19328, Jordan; a.khaled@ammanu.edu.jo; 5 Basic Pharmaceutical Science Department, Faculty of Pharmacy, Middle East University, Amman 11831, Jordan; d.abudalo@meu.edu.jo; 6 Institute of Nanotechnology, Jordan University of Science and Technology, Irbid 22110, Jordan; aabouzieh@just.edu.jo; 7 Department of Nanoscience, Joint School of Nanoscience and Nanoengineering, University of North Carolina at Greensboro, Greensboro, NC 27401, USA; raafaneh@uncg.edu

**Keywords:** mixed-matrix membranes, coated membranes, photocatalytic membrane, plasma treatment, flux

## Abstract

Polyethersulfone (PES) membranes are essential in separation processes; however, their inherent hydrophobicity can limit their effectiveness in water-intensive applications. This study aims to enhance PES membranes by modifying them with a NiFe_2_O_4_–nanoclay composite nanoparticle to improve both their hydrophilicity and photocatalytic potential as a photocatalytic membrane. The nanoparticles were synthesized using the sol–gel auto-combustion method and incorporated into PES membranes through mixed-matrix embedding (1 wt% and 3 wt%) and surface coating. X-ray diffraction confirmed the cubic spinel structure of the composite nanoparticles, which followed the second order kinetic reaction during the photodegradation–adsorption of crystal violet. The mixed-matrix membranes displayed a remarkable 170% increase in water flux and a 25% improvement in mechanical strength, accompanied by a slight decrease in contact angle at 1 wt% of nanoparticle loading. In contrast, the surface-coated membranes demonstrated a significant reduction in contact angle to 18°, indicating a highly hydrophilic surface and increased roughness. All membranes achieved high dye removal rates of 98–99%, but only the coated membrane system exhibited approximately 50% photocatalytic degradation, following mixed kinetics. These results highlight the critical importance of surface modification in advancing PES membranes, as it significantly reduces fouling and enhances water–material interaction qualities essential for future filtration and photocatalytic applications. Exploring hybrid strategies that combine both embedding and coating approaches may yield even greater synergies in membrane functionality.

## 1. Introduction

Water scarcity is a major global problem that is worsening due to population growth and industrialization. The most significant impact is the growing water demand, combined with a decreasing supply, which affects the amount of water available to each person. As a result, around a billion people worldwide lack access to safe drinking water. This makes it crucial to find solutions to address the issue [1,2]. One key approach in environmental studies is treating contaminated water. However, these methods may not be effective enough due to the severity of water pollution, which includes long-lasting pollutants, heavy metals, and new contaminants such as pharmaceuticals and personal care products. Additionally, treatment processes often have inefficiencies [3].

Given this context, it is clear that developing advanced methods with high separation efficiency and operational flexibility is crucial. Polymeric membranes, particularly polyethersulfone (PES), stand out for their high flexibility, chemical strength, and exceptional thermal stability [4,5]. A key benefit of these membranes is their resistance to harsh operating conditions, making them suitable for various water treatment applications, from ultrafiltration to nanofiltration [6]. However, there is an urgent need to modify these membranes to achieve higher purification levels, particularly in fouling resistance and catalytic properties [7,8].

Nanomaterials find diverse applications across multiple fields, ranging from antibacterial agents [9] to advanced detection techniques [10]. Due to their impressive capabilities, researchers have focused on nanomaterials for their potential in membrane technology. Recently, numerous studies have investigated methods for integrating materials such as titanium dioxide (TiO_2_), graphene oxide (GO), and zinc oxide (ZnO) into membranes. The goal is to boost their photocatalytic, antimicrobial, and mechanical properties [11,12]. By modifying these membranes, researchers can not only physically separate contaminants but also break them down through advanced oxidation processes. This tackles a major limitation of traditional membrane filtration, which only concentrates contaminants rather than destroying them [13].

To keep up with recent advancements in this field, it is crucial to explore more efficient nanostructures. Nickel ferrite (NiFe_2_O_4_), a magnetic spinel oxide, shows great potential as a photocatalytic material due to its narrow band gap, excellent magnetic properties, and remarkable chemical stability [14]. It holds great potential for biosensing uses, given that its surface readily allows for the attachment of biomolecules and recording materials and is utilized in various fields, such as aviation, medication delivery, dyes, and sensors [15,16,17,18]. On the other hand, nanoclay materials have found widespread use in various applications because of their ability to serve as support materials and their unique properties, which enhance interactions between different surfaces. Furthermore, they are hydrophilic, especially when combined with polymeric matrices [19,20]. Combining two compounds, such as nanoclay and NiFe_2_O_4_, into a composite material presents a promising strategy for developing next-generation multifunctional membranes that offer improved photocatalytic efficiency and separation capabilities [21].

This study presents two approaches to the fabrication of membranes, marking the first investigation into the integration of NiFe_2_O_4_–nanoclay composites with PES membranes. The techniques employed are the mixed-matrix membrane methodology, which allows for bulk incorporation of the composite material, and the dip coating method, used for surface functionalization.

The primary goal of this study is to identify the best approach for developing and characterizing PES-NiFe_2_O_4_–nanoclay membrane systems. We aim to examine their structural and morphological characteristics and how these factors influence key outcomes, including water permeability, rejection performance, and photocatalytic activity. Furthermore, this research seeks to enhance scientific understanding of nanoparticle systems while offering practical insights for the development of more efficient water treatment techniques.

## 2. Materials and Methods

### 2.1. Materials

For nanoparticle synthesis: iron(III) nitrate nonahydrate Fe(NO_3_)_3_·9H_2_O M = 404.00 g/mol; nickel(II) nitrate hexahydrate Ni(NO_3_)_2_·6H_2_O M.W.: 290.79, green crystals; tartaric acid (C_4_H_6_O_6_); and modified nanoclay surface containing 25–30 wt% trimethyl stearyl ammonium (Montmorillonite clay) (Sigma-Aldrich, St. Louis, MO, USA).

For membrane preparation: poly(ether-sulfone) (PES) granule (Mw 58,000 g/mol, Goodfellow, England); polyvinylpyrrolidone (PVP) with an average MW of 36,000 (Sigma-Aldrich, Beijing, China); 1-methyl-2-pyrrolidinone (NMP) (AppLiChem, Ottoweg 4 D-64291 Darmstadt, Germany); Crystal Violet and distilled water.

### 2.2. Nanoparticle Synthesis

Using the sol–gel auto-combustion method, the NiFe_2_O_4_–nanoclay nanocomposite was synthesized. Tartaric acid (C_4_H_6_O_6_) served as both a chelating agent and a fuel. Nickel nitrate (Ni(NO_3_)_2_·6H_2_O) (17.239 g) and iron nitrate (Fe(NO_3_)_3_·4H_2_O) (6.201 g) were employed as metal precursors, mixed in stoichiometric proportions (1:2). Next, a tartaric acid solution (28.18 g) was added to the metal nitrate mixture at a molar ratio of 3:1 (tartaric acid to total metal cations). Nanoclay was incorporated into the metal nitrate solution at a weight ratio of 3:1 (clay to ferrite), and the mixture was held at 75 °C in a water bath under continuous stirring at 330 rpm. The resulting reaction mixture was heated until a gel formed. This gel was subsequently heated on a hot plate, with the temperature gradually increasing to 350 °C, at which point auto-combustion occurred, yielding a fine powder. The powder was then subjected to thermal treatment through calcination in a furnace at 500 °C for 5 h to complete the synthesis [22].

### 2.3. Nanoparticle Characterization

#### 2.3.1. Nanoparticle Structure

X-ray diffraction (XRD) analysis was conducted using a Rigaku Ultima IV instrument from Tokyo, Japan, in which a Cu K X-ray tube at 20 mA and 40 kV, with a scan speed of 3000°/min and a step size of 0.02 were used. The analysis covered a range from 3° to 90°. Additionally, nanoparticle size and shape were examined using a Quanta scanning electron microscope (SEM) in Eindhoven, The Netherlands.

#### 2.3.2. Nanoparticles’ Photocatalytic Activity

The photocatalytic performance of the synthesized nanoparticles was evaluated in a batch system under a commercial visible metal halide lamp (HQI-T250/Daylight, OSRAM GmbH, Munich, Germany) with a luminous efficacy of 82 lm/W and a luminous flux of 20,000 lm for irradiation (wavelengths 380–780 nm), where the degradation of Crystal Violet was measured. The contact time was examined at a neutral pH of 7, using a nanoparticle concentration of 0.5 g/L and three varying initial concentrations of Crystal Violet (CV): 20, 30, and 40 ppm. The contact time was changed from 0 to 50 min to assess the efficiency of degradation over time. Kinetic analyses were also performed to comprehend the degradation dynamics.

**Scheme 1:**
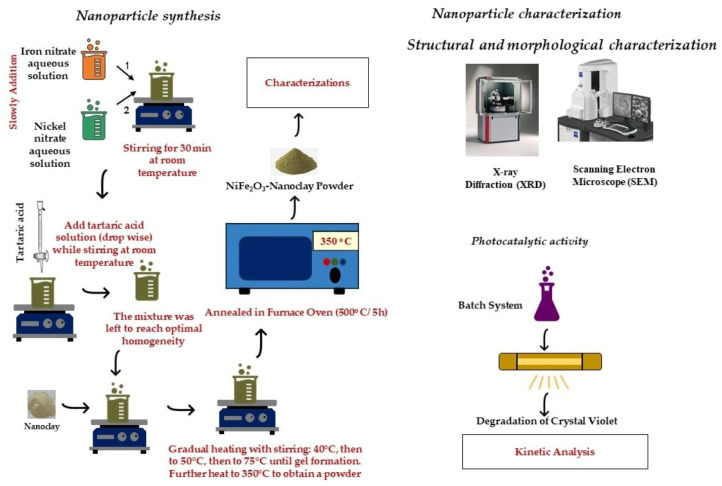
NiFe_2_O_4_–nanoclay synthesis and characterization.

Details of nanoparticle synthesis and characterization are shown in Scheme 1.

### 2.4. Membrane Systems Preparation

#### 2.4.1. Mixed-Matrix Membranes

The pure membrane (without nanoparticles) was prepared by dissolving PES (18 wt%) and PVP (1 wt%) in NMP (81 wt%) as the solvent. The mixture was stirred at 60 °C overnight to obtain a casting solution. This solution was then used to fabricate membranes using the phase inversion method. For the mixed-matrix membranes, concentrations of 1 wt% and 3 wt% NiFe_2_O_4_–nanoclay were incorporated. Specifically, 0.11 g and 0.33 g of the NiFe_2_O_4_–nanoclay nanocomposite were gradually dispersed into NMP and sonicated for 30 min to ensure uniform dispersion. Following this, the appropriate amounts of PES and PVP were added to the (NiFe_2_O_4_–nanoclay)–NMP dispersion, and the solution was stirred continuously at 60 °C overnight. The resulting mixtures were then cast into membranes using the phase inversion method. An average thickness of the prepared membranes was 230 µm, and three samples of each membrane type were prepared.

#### 2.4.2. Coated Membranes

For the coating solution, 0.3 g of NiFe_2_O_4_–nanoclay were dissolved in 1 L of distilled water, followed by 30 min of sonication to ensure good dispersion [2]. A pure PES membrane (containing 0% NiFe_2_O_4_-Nanoclay) was used. To enhance surface adhesion, the membrane underwent plasma treatment for 30 min before being dip-coated in the prepared NiFe_2_O_4_–nanoclay solution. The plasma-treated membranes were then immersed in the solution for an additional 30 min. After the coatings were applied, the membranes were removed from the solution and dried in an oven at 50 °C for 10 min, making them ready for the photocatalytic performance experiment.

**Scheme 2:**
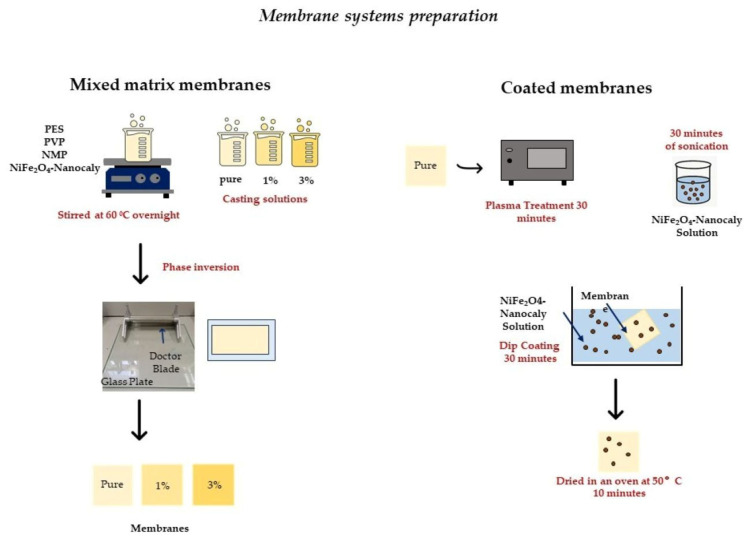
The preparation of mixed-matrix membranes and coated membranes.

The preparation of the membrane systems is shown in Scheme 2.

### 2.5. Membrane Systems Characterization

#### 2.5.1. Surface Morphology and Structure Properties

Membrane solution viscosities were tested using a FUNGILAB viscometer (Barcelona, Spain) with rod L2. An SEM from Quanta FEG 450, Eindhoven, The Netherlands, was used to examine the membranes’ surface morphology. Contact angle was used to evaluate the hydrophilicity of the membranes’ surfaces using Biolin Scientific’s Attension (Västra Frölunda, Sweden) with the sessile drop technique. The membranes’ surface topologies were examined using Atomic Force Microscopy (AFM) (AIST-NT SmartSPM 1000) (Moscow, Russia), which functioned in non-contact mode using NSC14/Al BC. The SI tips were operated at 160 kHz resonant frequency, with a scanning rate that varied between 0.3 kHz and 1 kHz. XRD analysis was conducted using a Cu K X-ray tube at 30 mA and 40 kV, with a scan speed of 3000°/min and a step size of 0.02.

#### 2.5.2. Barrier Properties

Utilizing a dead-end cell (HP4750; Sterlitech, Auburn, WA, USA), measurements of the pure water flux across membranes with an area of 14.6 cm^2^ at a pressure of 1.0 bar were conducted. The permeability flux (J_w_) was determined using Equation (1):

(1) *J_w_* = *vA* · Δ*T*

where v (L) denotes the volume of water permeate, A (m^2^) signifies the effective membrane area, and ΔT (h) reflects the permeability time.

Rejection rate was measured using a UV–Vis spectrophotometer to determine the concentration of Crystal Violet before and after filtration by the dead-end cell.

#### 2.5.3. Mechanical Properties

Tests were run to evaluate the tensile properties of the membrane using a BMT-E Series testing machine from BESMAK in Ankara, Turkey. The tests were conducted at a tensile speed of 5 mm/min.

#### 2.5.4. Photocatalytic Properties

The photocatalytic degradation experiment was performed on pure PES, mixed-matrix membranes (1 wt% NiFe_2_O_4_–nanoclay), and coated mixed-matrix membranes. Following the procedure of a batch system adopted by Popa et al. [23], a 2.5 × 2.5 cm^2^ membrane sample was immersed in a beaker containing 15 mL of 10 ppm Crystal Violet aqueous solution under continuous stirring. The system was first kept in darkness for 30. Subsequently, the solution was irradiated using a commercial visible metal halide lamp (HQI-T250/Daylight, OSRAM GmbH, Germany) with a luminous efficacy of 82 lm/W and luminous flux of irradiation 20,000 lm (wavelengths 380–780 nm). The photocatalytic degradation process was conducted for 2 h, during which 1 mL aliquots were collected at 30 min intervals for subsequent analysis. This systematic approach allowed for the evaluation of the photocatalytic performance across all membrane variants under controlled conditions.

## 3. Results and Discussion

### 3.1. Nanoparticle Structure Characterization

#### 3.1.1. XRD

**Figure 1:**
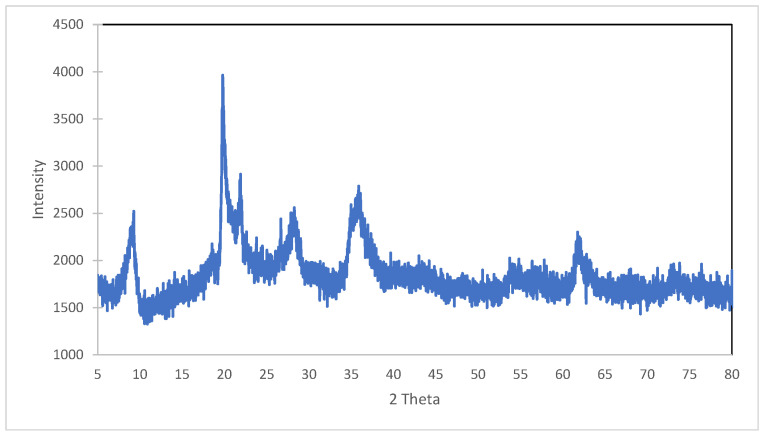
XRD pattern of NiFe_2_O_4_–nanoclay.

The XRD pattern of the NiFe_2_O_4_–nanoclay composite, shown in Figure 1, displays distinct peaks, each providing insights into the material’s structural characteristics. Notably, the peak at 36° corresponds to the (311) plane of the cubic spinel structure of NiFe_2_O_4_, which is typically the most intense reflection, confirming that the ferrite phase formed successfully. Similarly, the peaks at 54°, 62°, 74°, and 77° align with the (422), (440), (533), and (444) planes of NiFe_2_O_4_, validating the cubic spinel crystalline structure, consistent with the diffraction card JCPDS 54–0964 [24,25,26]. The peak at 9° may indicate an interlayer spacing characteristic of layered silicates in surface-modified nanoclay. Peaks at 20°, 27°, 36°, and 54° correspond to the planes (110), (210), (124), and (144). Overall, the XRD pattern suggests a successful integration of NiFe_2_O_4_ within the nanoclay matrix, with residual signatures of plant-derived organics.

#### 3.1.2. SEM

**Figure 2:**
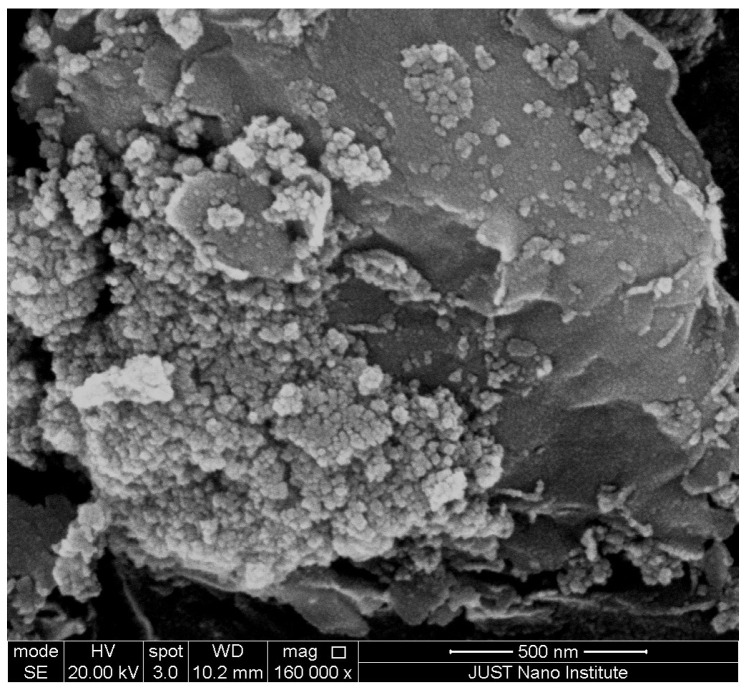
SEM image of the nanoparticle composite (NiFe_2_O_4_–nanoclay), at magnification of 160,000. The scale bar = 500 nm.

Figure 2 depicts the structure of the synthesized nanoparticle composite (NiFe_2_O_4_–nanoclay). The oxide particles displayed are nearly spherical and are unevenly distributed across the nanoclay platelets, showing some signs of agglomeration, with an average size of 30 nm, as determined using Jmicrovidion software 1.3.4. This uneven distribution and the agglomeration of nanoparticles arise from strong magnetic interactions, Van der Waals forces, and their high surface energy, as noted by Baul et al., 2023 [25], who reported similar findings.

### 3.2. Photocatalytic Activity of Nanoparticles

**Figure 3:**
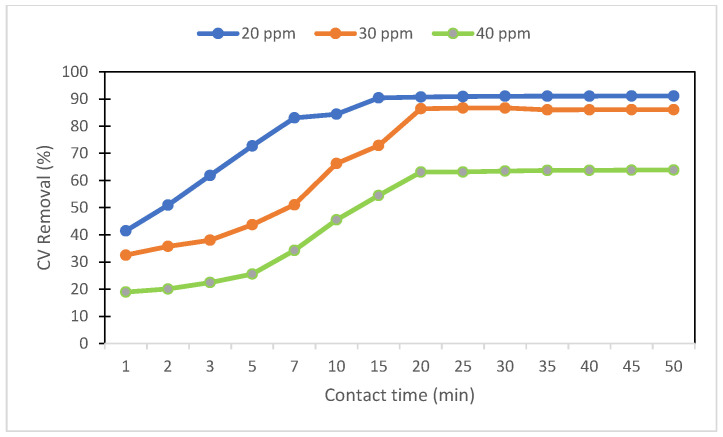
Contact-time photocatalysis experiment at pH 7 and a nanoparticle dose of 0.5 g/L.

Figure 3 illustrates the photocatalytic degradation of Crystal Violet (CV) at different initial dye concentrations, employing the synthesized NiFe_2_O_4_–nanoclay composite. The photocatalyst demonstrated a rapid degradation activity, with the reaction nearing equilibrium within 15 to 20 min. Notably, the rate of degradation and the time required to achieve equilibrium were inversely related to the initial CV concentration. At lower concentrations, particularly at 20 ppm, the photocatalyst showed significantly enhanced activity, reaching approximately 90% degradation in just 15 min. This can be attributed to the greater availability of active sites on the catalyst’s surface relative to the number of dye molecules at lower concentrations, leading to faster adsorption and subsequent photocatalytic breakdown.

In comparison to the literature, NiFe_2_O_4_, ZnO/NiFe_2_O_4,_ and ZnO/NiFe_2_O_4_-rGO photocatalysts were synthesized by Ihsan et al. (2022), who tested their activity of photocatalytic degradation on CV, and found that it degraded CV by 41.9, 43.4, and 80.5% after 140 min [27].

To investigate the reaction kinetics of Crystal Violet (CV) degradation over the NiFe_2_O_4_–nanoclay photocatalyst, the experimental data were analyzed using kinetic models of zero-, first-, and second-order reactions. The linearized forms of each kinetic equation were applied to the degradation data, and the corresponding plots were generated by fitting concentration (or ln(C) and 1/C) against time. The linearity of each plot, indicated by the R^2^ values, was evaluated to determine the best-fitting model. This analysis provided insight into the underlying reaction mechanism and the influence of dye concentration on degradation behavior, enabling a deeper understanding of how these factors interact during the photocatalytic process.

**Figure 4:**
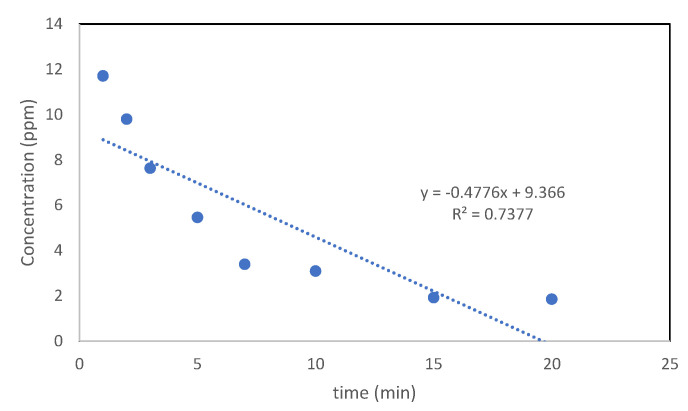
Zero-order kinetics for photocatalytic degradation at pH 7, a nanoparticle dose of 0.5 g/L, and initial CV concentration of 20 ppm.

**Figure 5:**
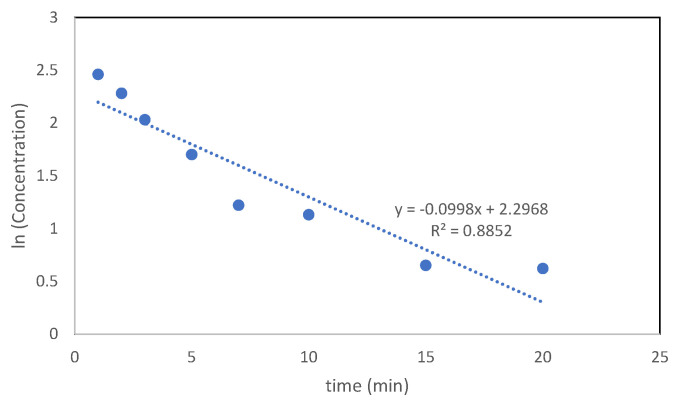
First-order kinetics for photocatalytic degradation at pH 7, a nanoparticle dose of 0.5 g/L, and initial CV concentration of 20 ppm.

**Figure 6:**
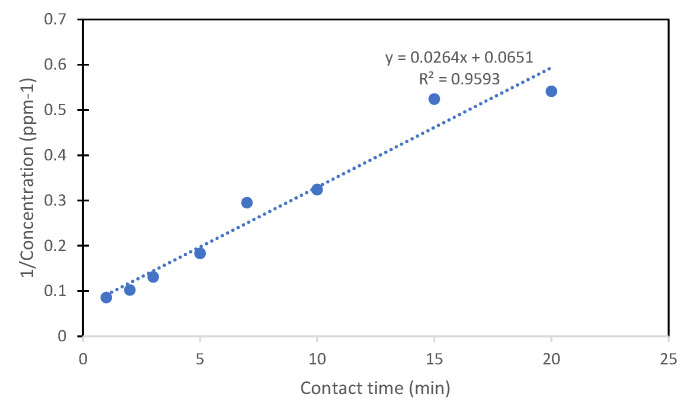
Second-order kinetics for photocatalytic degradation at pH 7, a nanoparticle dose of 0.5 g/L, and initial CV concentration of 20 ppm.

Figure 4, Figure 5 and Figure 6 present the linearized plots for zero-, first-, and second-order kinetic models related to the photocatalytic degradation of Crystal Violet using the NiFe_2_O_4_–nanoclay composite. Among these models, the second-order plot shows the best linearity, with a correlation coefficient (R^2^) of 0.9593. From Figure 5, the rate constant K = 0.0651 L/mg·min. This indicates that the degradation process adheres to second-order kinetics, and that the photocatalytic process is likely governed by chemisorption-driven surface interactions, where the pollutant molecules are strongly adsorbed onto the nanoparticle surface prior to degradation. Such behavior supports the involvement of active sites in facilitating charge transfer during the photocatalytic reaction. In this context, the rate of degradation depends on both the dye concentration and the availability of active sites on the surface of the photocatalyst, which is typical of surface-mediated reactions.

### 3.3. Characterization of Membranes

#### 3.3.1. Membrane Solution Viscosity

The viscosity of each membrane casting solution was measured to assess the influence of nanoparticle loading on the rheological properties of the dope solutions. The results revealed that the viscosity values were 310.0 ± 5.7 mPa·s for the pure PES solution, increasing to 410.2 ± 5.7 mPa·s for the 1 wt% NiFe_2_O_4_–nanoclay composite-loaded PES solution, and then decreasing significantly to 150.1 ± 0.7 mPa·s for the 3 wt% loaded solution.

The initial increase in viscosity at 1 wt% loading suggests improved interaction and homogeneous dispersion of the nanoparticles within the polymer matrix, likely due to good compatibility and favorable interfacial interactions between the filler and the polymer chains. However, the unexpected drop in viscosity at 3 wt% could be attributed to nanoparticle agglomeration at higher concentrations, leading to poor dispersion and possibly disrupting the entanglement of polymer chains.

#### 3.3.2. SEM

**Figure 7:**
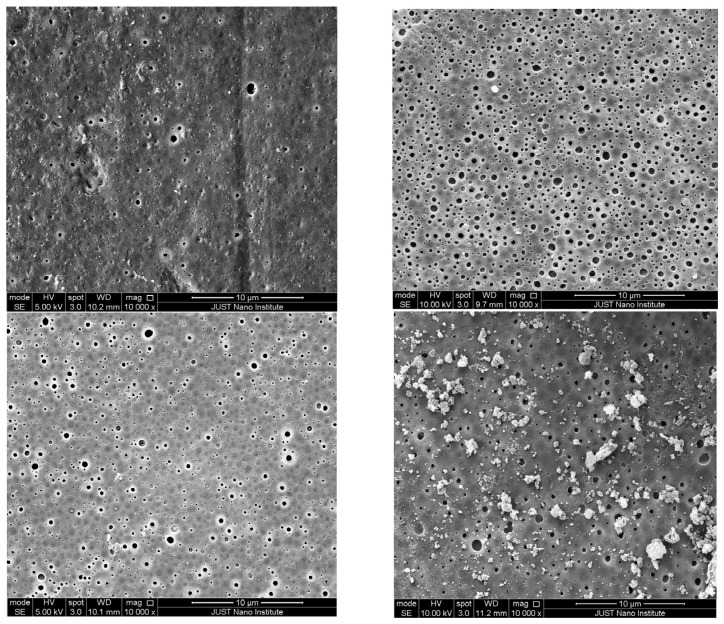

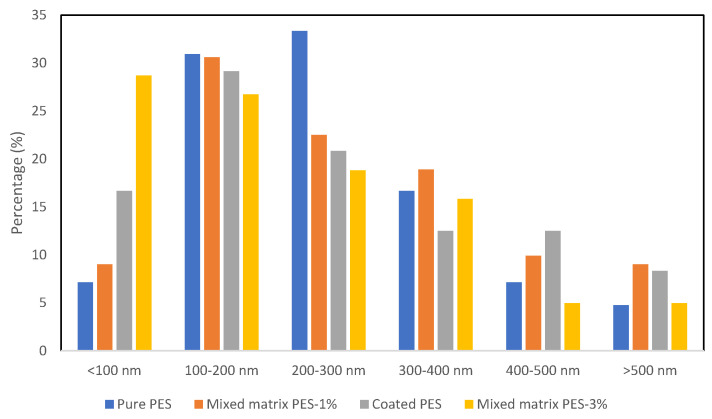
SEM images of the surface of pure PES, mixed-matrix PES (1 wt% and 3 wt%), and coated PES, respectively, at a magnification of 10,000. Scale bar = 10 µm; pore-size distribution histogram.

Figure 7 illustrates the surface images of three membrane systems: pure PES, mixed-matrix PES, and coated PES. The pore size was measured using Jmicrovision software, revealing average diameters of (261 ± 160) nm, (271 ± 155) nm, (189 ± 130), and (258 ± 166) nm, respectively, and a histogram of pore size distribution is shown, indicating that the introduction of nanoparticles into the polymer matrix did not significantly affect the overall pore size, except for the 3 wt% mixed-matrix membrane, which exhibited a lower average pore size. This can be attributed to the higher concentration of nanoparticles. This increased concentration limits the mobility of the polymer chains, leading to a denser packing arrangement. As a result, the pore sizes between the polymer chains are reduced, affecting the overall properties of the membrane. This phenomenon highlights the impact of nanoparticle concentration on the structure and functionality of mixed-matrix membranes. It was also noted that the pore size distributions of all samples were similarly broad, with coefficients of variation around 60%, indicating inconsistent pore structure across samples. This indicates a lack of control over pore formation, leading to non-uniform and inconsistent pore structures regardless of the membrane modification method. However, it is evident that the mixed-matrix membranes exhibited a substantial increase in the number of pores. This can be attributed to the introduction of hydrophilic nanoparticles into the casting solution, which enhances the exchange rate between solvents and non-solvents during the phase inversion process. The presence of these nanoparticles facilitates water diffusion into the polymeric film, thereby promoting pore formation and improving overall porosity [28,29,30]. Conversely, the size of the NiFe_2_O_4_–nanoclay composites distributed on the PES surface was measured and found to have an average size of (418 nm ± 282 nm), which represents the width of the nanoclay platelets. These measurements highlight the characteristics of the nanocomposite structure and its potential impact on the membrane’s performance.

These observations can be better understood by considering the underlying mechanisms governing membrane formation. The observed morphological changes result from the interaction between nanoparticle-induced thermodynamic and kinetic effects during phase inversion. Adding hydrophilic NiFe_2_O_4_–nanoclay composites modifies the solvent–non-solvent exchange by increasing the polymer–solvent affinity and boosting local osmotic pressure gradients. This encourages immediate demixing, speeds up pore nucleation, and helps form voids, especially in mixed-matrix membranes. Furthermore, the surface-localized nanoparticles in coated membranes are not expected to disrupt the bulk pore architecture, supporting the trend of comparable average pore sizes across systems.

#### 3.3.3. XRD

**Figure 8:**
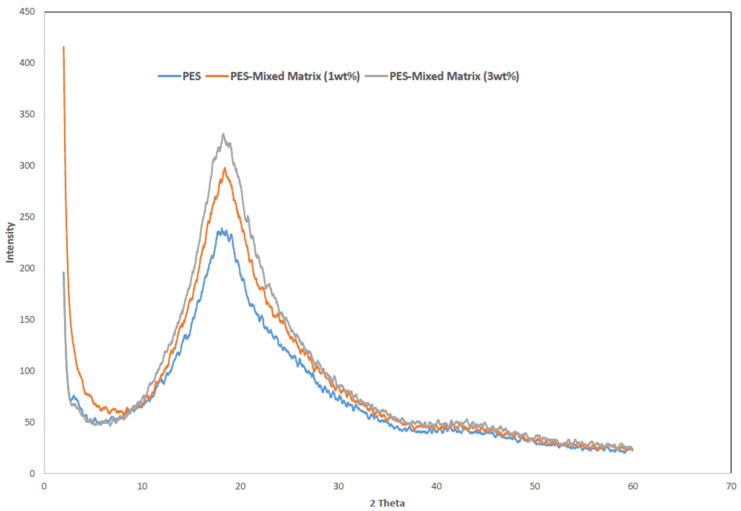
XRD patterns for PES membrane systems.

X-ray diffraction (XRD) analysis was performed on the PES membrane systems to explore how the incorporation of nanoparticles affects their structure (Figure 8). The findings showed that adding NiFe_2_O_4_–nanoclay composite nanoparticles into the polymer matrix had a significant impact on the membranes’ crystallinity. This was demonstrated by the increased intensity and sharper diffraction peaks, especially at higher nanoparticle concentrations. The improved crystallinity is likely due to the nanoparticles acting as nucleating agents, facilitating a more organized alignment of polymer chains during membrane formation. As the concentration of nanoparticles rises, these nucleation effects become more significant, resulting in better packing of polymer chains and the formation of crystalline domains. This increase in crystallinity is anticipated to enhance the mechanical stability and modify the transport properties of the membrane.

Although XRD shows increased crystallinity with more nanoparticles, the viscosity of the casting solutions reveals the underlying mechanism. At 1 wt%, viscosity rises, indicating good dispersion and strong nanoparticle–polymer interaction, promoting uniform crystalline domains. At 3 wt%, viscosity drops, suggesting agglomeration and weaker interactions. Despite this, crystallinity rises, as the higher nanoparticle content offers more nucleation sites, with clusters inducing localized polymer alignment, increasing overall crystallinity, though less uniformly. Thus, reduced dispersion at higher loadings still increases crystallinity due to more nucleation sites.

#### 3.3.4. Surface Roughness and Hydrophilicity

The addition of nanoparticles to the PES membrane system, either as a mixed matrix or as a dip-coating, is expected to affect the surface roughness and chemistry, which must influence the membrane performance. Table 1 shows the data for surface roughness extracted from AFM and contact angle results. The influence of nanoparticle incorporation on surface roughness and wettability arises from the spatial distribution, surface energy, and interfacial interactions between the nanoparticles and the polymer matrix. The surface roughness for the mixed-matrix PES membrane at 1 wt% loading is almost the same as that of pure PES, which is due to the fact that the nanoparticles are distributed within the polymer matrix, and, as shown in the SEM image in Figure 7, no nanoparticles are distributed on the surface. In contrast, at 3 wt% loading, the surface roughness increased to 21.9 nm, which may indicate that some of the nanoparticles at this higher loading are distributed near or at the surface, as crowding near the surface becomes more probable due to saturation effects and kinetic constraints during film formation, leading to slight protrusions that elevate surface roughness. On the other hand, the surface roughness of the coated membranes is slightly increased from 15.74 nm to 17.03 nm, due to presence of the nanoparticles on the surface. The minimal increase in surface roughness can be attributed to the low concentration and small size of the nanoparticles (thickness), as well as their good compatibility with the membrane matrix. When the nanoparticles are well dispersed, and the coating thickness is low, they tend to fill surface valleys rather than create new peaks, resulting in only a slight change in the overall topography. Membrane coatings have been shown to have varying effects on surface topography in the literature. Zhang et al. (2017) [31] reported a significant increase in roughness after coating nanofiltration membranes with graphene oxide quantum dots (GO QDs). This increase was attributed to the formation of surface protrusions caused by the deposited nanoparticles [31]. In contrast, Choi et al. (2013) [32] observed a decrease in surface roughness when polyamide membranes were coated with graphene oxide nanosheets through a layer-by-layer assembly technique. In this case, the GO layers filled in surface valleys, resulting in a smoother membrane topology [32]. Compared to these studies, the minor topographical changes observed in our membranes indicate the effective integration of nanoparticles, which modify the surface moderately, without creating significant new features. The contact angle was not significantly affected in the mixed-matrix membranes; however, it was significantly decreased in the case of the coated membranes, which is expected due to the exposure of hydrophilic functional groups on the nanoparticle surfaces, which enhance surface polarity and increase hydrogen bonding interactions with water. In contrast, mixed-matrix membranes show limited change in contact angle because the hydrophilic components are mostly embedded and not in direct contact with the membrane–liquid interface.

#### 3.3.5. Permeability and Barrier Properties

**Figure 9:**
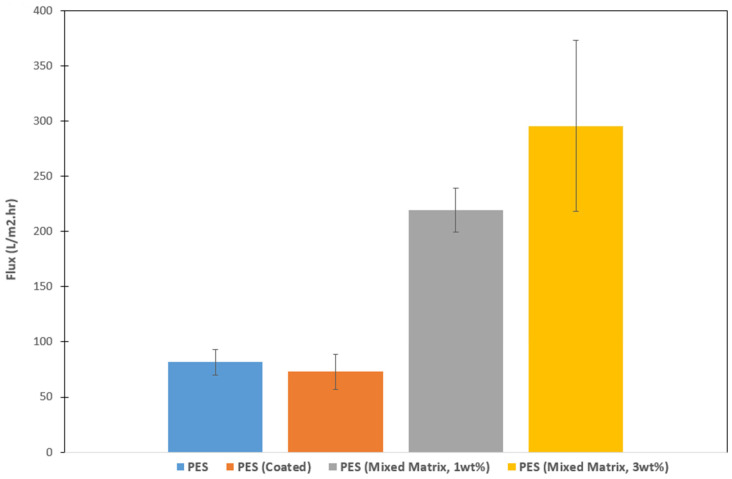
Water flux across PES membrane systems.

**Figure 10:**
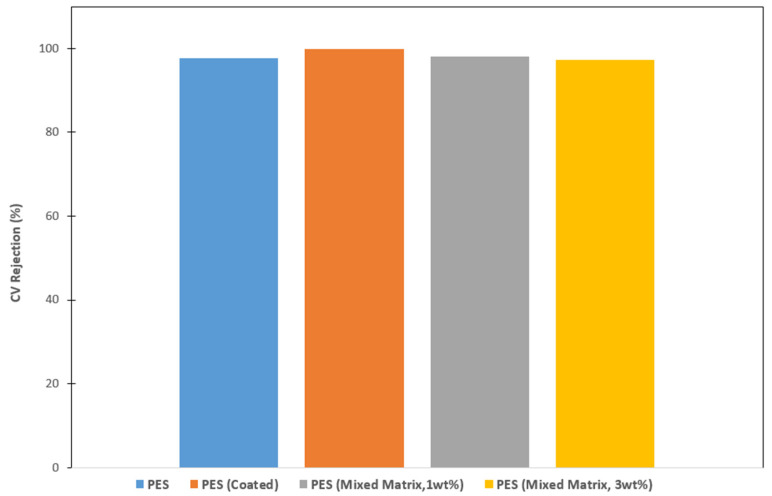
Crystal Violet dye rejection through PES membrane systems.

Permeability tests were conducted on all PES membrane systems to examine the effect of adding nanoparticle composites to the PES membrane, either within the polymer matrix or through coating. As shown in Figure 9, water permeability was not affected when the PES membranes were coated. It was initially expected that some of the nanoparticle composites would block the pores; however, since the coating only affects the surface, the porous structure throughout the thickness of the coated membranes was sufficient to retain the same permeability. In contrast, the water flux across the mixed-matrix PES membranes was enhanced by 170%, increasing from 81 L/m^2^·hr for the pure PES membranes to 219 L/m^2^·hr for the mixed-matrix PES membranes. This improvement can be attributed to the significant increase in porosity observed in the mixed-matrix membranes, as demonstrated in the SEM images (Figure 7). Additionally, the hydrophilic character of the nanoclay’s residual –OH groups and quaternary ammonium moieties may have increased membrane wettability, further contributing to higher flux. On the other hand, the flux of the 3 wt% mixed matrix was increased to 295 L/m^2^.hr; however, as seen from the error bar, there was a significant fluctuation in flux data, which indicated non-homogeneity of the distribution of the nanoparticles within the polymer matrix. This non-homogeneity could cause localized aggregation, as also confirmed by the viscosity results presented in Section 3.3.1, leading to uneven pore formation and varied transport pathways across the membrane surface. Furthermore, the high rejection rate of the Crystal Violet dye remained unchanged in all membrane systems (Figure 10). This was expected, as the pore size did not change with the inclusion of the NiFe_2_O_4_–nanoclay composite, whether incorporated within the PES polymer matrix or applied via dip-coating, as indicated by the SEM images in Figure 7. Therefore, the separation mechanism remained dominated by size exclusion and was not adversely affected by nanoparticle incorporation, whether via coating or embedding.

The observation that coating PES membranes with nanoparticle composites did not significantly affect water permeability aligns with previous studies. For instance, coatings with graphene oxide quantum dots have been shown to enhance water permeability and dye rejection simultaneously, without causing pore blockage, since the coating primarily modifies the membrane surface while preserving the underlying porous structure, in addition to enhancing surface chemistry [31]. Similarly, physically coated Cu-BTC/PES mixed-matrix membranes demonstrated high dye separation performance without compromising permeability, supporting the notion that surface coatings maintain effective porosity [33]. In contrast, the significant increase in water flux observed in the mixed-matrix PES membranes is consistent with findings in the literature, which suggest that incorporating nanoparticles into the polymer matrix enhances both membrane porosity and permeability. For example, the addition of nanoparticles such as sulfated TiO_2_ to PES membranes has been shown to improve water flux by increasing the porosity of the membrane [34].

#### 3.3.6. Mechanical Properties

**Figure 11:**
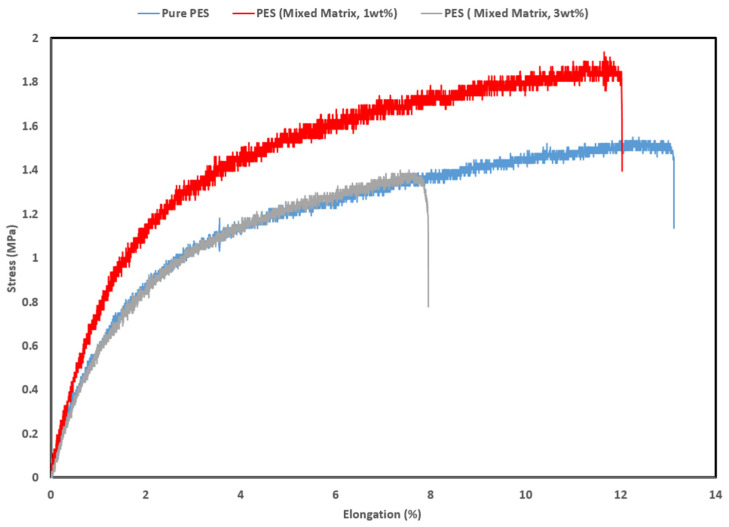
Mechanical properties of pure PES membranes and mixed-matrix PES membranes.

The inclusion of nanoparticle composites within the polymer matrix is expected to affect its mechanical properties. Figure 11 confirms that the addition of nanoparticle composites at 1 wt% enhanced the maximum tensile strength by 25%, while the tensile modulus was increased from 70 MPa in the pure PES to 90 MPa in the mixed-matrix PES (1 wt%), with an enhancement of 28.5%. The well-dispersed NiFe_2_O_4_–nanoclay particles likely acted as efficient stress-transfer centers within the polymer matrix, enhancing load distribution and increasing resistance to deformation under tensile stress. The interfacial adhesion between the polar functional groups on the modified nanoclay surface (e.g., –OH, quaternary ammonium) and the PES chains may have facilitated strong interfacial interactions, reducing interfacial slippage and enhancing the stiffness (modulus) of the resulting composite. However, the elongation at break decreased with the addition of nanoparticles. This reduction is due to the intrinsic brittleness of nanoclay, which restricts the mobility of polymer chains and diminishes the material’s ability to deform under stress. Similar results were observed with the addition of nanoclay and nanoclay composites [30,33,35,36]. On the other hand, the 3 wt% loading showed no enhancement in modulus or strength compared to the pure PES membrane, and lower tensile modulus and strength compared to the 1 wt% PES membranes, while the elongation decreased. This aligns with the conclusions drawn from the viscosity results and flux data fluctuations at 3 wt%, highlighting the non-homogeneity in the distribution of the nanoparticles, as well as some agglomeration. Agglomerates can serve as stress concentration points rather than reinforcements, initiating microcracks or interfacial debonding under tensile load. The combined mechanical and permeability behavior thus strongly supports the conclusion that nanoparticle dispersion and interfacial compatibility are critical determinants of nanocomposite membrane performance.

#### 3.3.7. Photocatalysis

**Figure 12:**
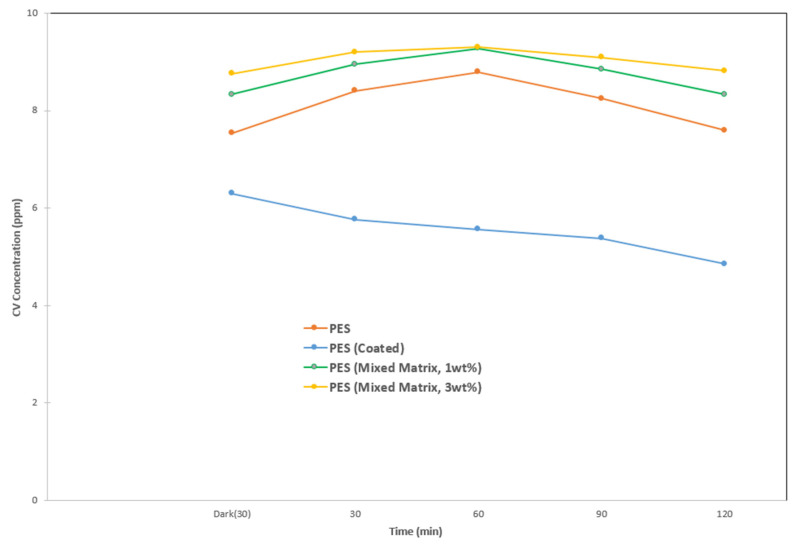
Photocatalysis experiment on membrane systems with an initial concentration of 10 ppm.

Figure 12 presents the photocatalytic performance of all membrane systems, including the pristine PES, mixed-matrix PES, and coated PES membranes. The results clearly show that only the coated membrane exhibits noticeable photocatalytic activity, while the pristine and mixed-matrix PES membranes display negligible or no degradation of the dye under the same conditions. This suggests that the photocatalytic functionality is primarily attributed to the surface coating, which likely contains the active photocatalyst material. The CV concentration reached 4.85 ppm (around 50%) after 2 h in the coated PES membranes, while it only reached 7.5 ppm and 8.3 ppm in the pure PES membrane and in the mixed-matrix PES membrane, respectively.

In this system, the NiFe_2_O_4_–nanoclay composite, when applied as a surface coating, is directly exposed to incident light and dissolved contaminants, enabling effective generation and utilization of reactive oxygen species (ROS), such as hydroxyl radicals (•OH) and superoxide anions (O_2_•^−^). These species are crucial for initiating the degradation of organic dyes such as Crystal Violet. In contrast, the mixed-matrix PES membranes, in which the photocatalyst is embedded within the polymer bulk, showed negligible photocatalytic activity. This is likely due to the shielding effect of the polymer matrix, which reduces light penetration and restricts the interaction between the catalyst and the dye molecules in the solution.

Nguyen et al. (2023) [37] coated PES membranes with CuO/TiO_2_ nanoparticle composite using plasma treatment, at different loadings. The CV degradation ranged from 30% for the 1 wt% nanoparticle concentration to a maximum of 60% for the 10 wt% nanoparticle concentration [37]. In another study, Zhao et al. (2023), prepared a ZnS/Ag_2_S@PES photocatalytic membrane, which reached pollutant photodegradation efficiency of ~70% for methylene blue (MB) and 58% for tetracycline [38].

**Figure 13:**
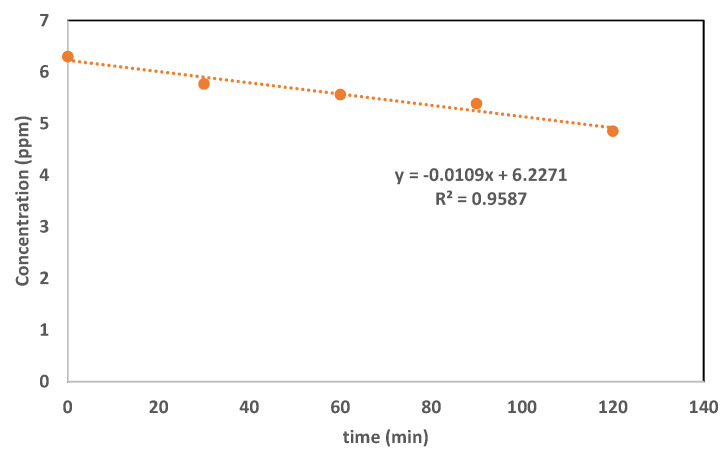
Zero-order kinetics for the photocatalytic degradation of the coated PES membrane.

**Figure 14:**
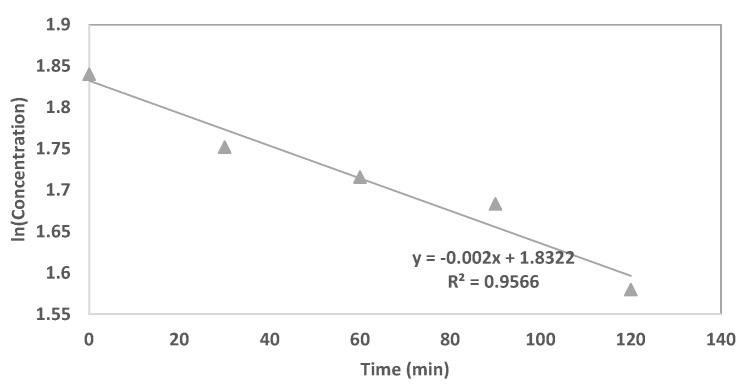
First-order kinetics for the photocatalytic degradation of the coated PES membrane.

**Figure 15:**
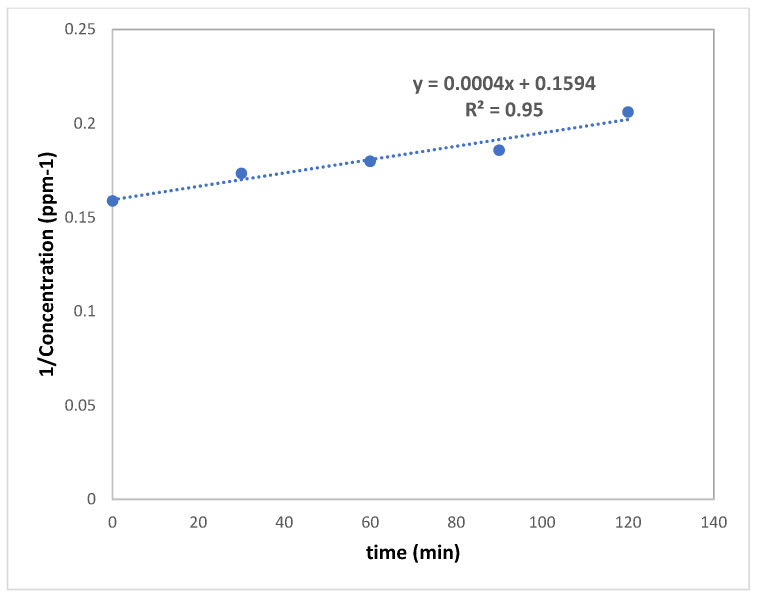
Second-order kinetics for the photocatalytic degradation of the coated PES membrane.

Figure 13, Figure 14 and Figure 15 illustrate the kinetic analysis of photocatalytic degradation using the coated PES membrane. The R^2^ values for the zero-, first-, and second-order models are 0.9587, 0.9566, and 0.9500, respectively, with rate constants of 0.0109 mg/L·min for the zero-order, 0.002 min^−1^ for the first-order, and 0.0004 L/mg·min for the second-order kinetics. The values indicate that the photocatalytic reaction occurs at a moderate rate and is likely driven by a combination of mechanisms, which include surface-limited reactions and processes that depend on concentration. The relatively high R^2^ value for the zero-order model suggests that, under the specified conditions, the degradation rate is probably more significantly impacted by factors such as the availability of active sites on the membrane surface, rather than the pollutant’s concentration, aligning with the surface-saturation behavior commonly seen in photocatalysis. However, the similar R^2^ values across all models indicate a complex mixed kinetic behavior that might involve several rate-determining steps. Mixed kinetics signifies a reaction mechanism that shifts between different kinetic orders based on operational conditions such as dye concentration, catalyst surface saturation, and mass transfer rates.

This complexity is highlighted when contrasting the coated membrane system with a suspended photocatalyst system, which exhibited a clear second-order kinetic behavior. The latter benefits from particle dispersion, unrestricted mass transfer, and maximum surface area exposure to light, allowing for more straightforward kinetics. In contrast, the immobilization of the photocatalyst on the membrane limits the accessibility of active sites, diminishes light absorption due to potential shadowing from the coating thickness, and introduces diffusion limitations within the membrane matrix. These factors lead to a heterogeneous reaction environment, whereby different regions or intervals of the process may exhibit distinct kinetic behavior. The transition from pure second-order kinetics to mixed kinetics illustrates the practical challenges faced in the membrane system and highlights the necessity for optimizing membrane design, ensuring uniform coating, and refining reactor conditions to improve photocatalytic efficiency.

## 4. Conclusions

The incorporation of NiFe_2_O_4_–nanoclay composite nanoparticles into polyethersulfone (PES) membranes markedly enhances their functional performance in water treatment applications, addressing multiple challenges typically faced by standard membranes. The use of mixed-matrix embedding and surface coating techniques each brings distinct advantages to the table. Specifically, the embedding process significantly improves the PES membranes’ mechanical strength and water permeability, enabling them to withstand high-pressure environments common in water treatment systems. The 1 wt% composite outperformed the 3 wt% composite due to better nanoparticle distribution and reduced agglomeration, enhancing interaction with the polymer matrix. Higher loadings can lead to clustering, which negatively impacts membrane performance. Thus, lower concentrations are more effective for high-performing membranes.

In contrast, the surface coating technique notably boosts hydrophilicity and enhances the membranes’ photocatalytic activity, an essential property for breaking down organic pollutants.

These enhancements tackle the significant limitations of pristine PES membranes, such as their inherent hydrophobicity, which often leads to fouling, diminishing efficiency and increasing operational costs. By improving separation efficiency and fouling resistance, these modified membranes extend their applicability in advanced systems such as photocatalytic membrane reactors and sophisticated filtration setups.

The promising synergistic potential of combining embedding and coating methods highlights a pathway for optimizing the overall architecture of the membranes. This optimization can lead to superior separation efficiency, fouling resistance, and even self-cleaning capabilities, which are invaluable for modern water treatment processes.

Looking ahead, possible applications of these innovative hybrid membranes include their integration into industrial wastewater treatment facilities and decentralized water purification systems. In both contexts, the demand for robust, high-flux membranes that exhibit low fouling characteristics coupled with photocatalytic functions is essential for ensuring effective water management. Furthermore, this hybrid membrane technology may facilitate the design of tailored multifunctional membranes capable of targeting a broader spectrum of organic pollutants and pathogens. This potential contributes significantly to sustainable water management initiatives and environmental protection efforts, aiming for cleaner water systems and reduced ecological impact.

## Figures and Tables

**Table 1:** Surface roughness and hydrophilicity.

Membrane	Surface Roughness (RMS) (nm)	Contact Angle (°)
PES	15.74	80
PES-1% Mixed matrix	15.53	75
PES-3% Mixed matrix	21.91	76
PES-Coated	17.03	18

## Data Availability

Data are contained within the article.
